# Parents’ experiences of infant and young child feeding during the COVID-19 pandemic in Ireland

**DOI:** 10.1017/S1368980023002343

**Published:** 2023-12

**Authors:** Elizabeth J O’Sullivan, Aileen Kennedy

**Affiliations:** School of Biological, Health and Sports Sciences, City Campus, Technological University Dublin, CQ312 Central Quad, Grangegorman, Dublin, Ireland

**Keywords:** Infant feeding, Child feeding, Breast-feeding, Formula feeding, Food security, Support, Breast milk substitutes

## Abstract

**Objective:**

The WHO has urged member states to develop preparedness plans for infant and young child feeding (IYCF) during emergencies. Ireland has no such plan. We aimed to identify the needs of caregivers in Ireland with regards IYCF during the COVID-19 pandemic.

**Design:**

Online survey conducted in May–June 2020.

**Setting:**

Ireland, during the first period of severely restricted movement due to COVID-19 (lockdown).

**Participants:**

Respondents (*n* 745) were primary caregivers of a child under 2 years; they were primarily well educated and likely of higher socio-economic status.

**Results:**

Among those who breastfed, being unable to access breast-feeding support groups and being unable to access in-person, one-to-one breast-feeding assistance were the biggest challenges reported. Nearly three quarters of those who had their babies during lockdown reported these challenges: 72·8 % and 68·8 %, respectively. For those using formula, the main challenges were structural in nature; approximately two-thirds of those who had their baby prior to lockdown feared there would be formula shortages and a third were unable to purchase formula due to shortages.

**Conclusions:**

Regardless of how their babies were fed, parents in Ireland experienced multiple challenges with infant feeding during the COVID-19 crisis. Breast-feeding should be protected, supported and promoted, particularly during an infectious disease pandemic. Additionally, assurances around supply of infant formula could reduce parental stress during a pandemic or emergency. An IYCF in emergencies plan would clearly set out how we could best support and protect the nutrition of the most vulnerable members of our population.

While the risks of not breast-feeding for both infants and mothers have been well documented^(1)^, breast-feeding is especially important in emergency situations, as infant feeding can easily be disrupted, leading to a risk of high mortality rates as result of respiratory and diarrhoeal infections and the associated risk of malnutrition^(2,3)^. A recent literature review has shown that disasters and emergencies are a major contributor to disruptions in breast-feeding^(4)^. While breast-feeding can ensure food security for vulnerable infants and young children, it should not be assumed that breast-feeding is something that women simply *do* during emergencies. Mothers need support to continue breast-feeding, as many women affected by emergencies may have seen their normal social support network severely disrupted and may be experiencing considerable stress^(5)^. During the COVID-19 pandemic, there was uncertainty among some about whether vertical transmission of the virus through breast milk was possible and some policies were introduced that led to the separation of mothers with confirmed or suspected COVID-19 and their babies^(6,7)^. However, the WHO^(8)^ and others^(9,10)^ have stated that continued breast-feeding is optimal for child health, even if the mother has a suspected or confirmed case of COVID-19.

Among infants consuming formula, international evidence shows frequent health consequences such as diarrhoea, infectious diseases and malnutrition associated with formula usage in emergencies^(4,11,12)^. In addition, food security for these infants is a concern, where possible interruptions to supply chains or healthcare accessibility or access to clean water and sanitation may occur, putting these infants and young children at additional risk^(13)^. During the recent COVID-19 pandemic, news outlets and individuals on social media reported families experiencing shortages of formula due to people purchasing bulk quantities of formula due to fears of forthcoming shortages both nationally in Ireland^(14)^ and internationally^(15)^. Therefore, caregivers of infants consuming formula require assistance to ensure continued access to the resources needed to feed their infants safety.

With global climate changes resulting in an increasing frequency of natural disasters, as well as the recent COVID-19 pandemic, it is essential for all countries to be prepared for any challenges with or interruptions to safe infant feeding^(16)^. The WHO recognises that infants and young children are among the most vulnerable victims of natural or human-induced emergencies and need vital support. As such, the WHO urged all member states to develop and implement a national Infant and Young Child Feeding in Emergencies (IYCF-E) preparedness plan^(16)^. Such a plan should integrate practices and programmes at a national level, in line with an international guidance plan developed by the IYCF-E Core Group^(17)^. This guidance document also recommends that individual jurisdictions should develop a context-specific action plan, as needs will vary from location to location.

While many countries have developed national emergency plans, few have included guidelines on safe infant and young children feeding. A recent audit of Australian emergency plans and guidance showed that consideration for how infants and young children’s needs could be met in emergencies was infrequently addressed by local or national agencies^(18)^. In the UK, the government has published guidance on evacuation and shelter, including advice on the care of vulnerable people, but there is no mention of mothers and infants^(19)^ and no UK-wide strategies addressing IYCF-E have been developed. The preparedness and planning for IYCF-E throughout Europe have been described as ‘seriously neglected’^(20)^.

Similarly in Ireland, a framework has been developed for the co-ordinated response to major emergencies^(21,22)^. While the framework issues guidance on the setting up of provision centres and advice on vulnerable sections of the community and their needs, no consideration has been given to unique needs of infants and young children, as they are not listed as vulnerable subgroups. The COVID-19 pandemic has highlighted that Ireland is vulnerable to a wide range of public health emergencies and has highlighted many deficiencies in our policy/planning.

Throughout the COVID-19 pandemic, there were significant changes to usual maternity care, with postnatal supports appearing to have been most affected^(23)^. The response to the pandemic led to the cancellation of breast-feeding support groups, which are often used by mothers as a social outlet and way to meet other new mothers. The response also led to the redeployment of public health nurses by the Health Service Executive, which delayed postnatal and developmental checks in the community, and restrictions in accessing GP and other healthcare professionals^(24)^. In light of anecdotal evidence of challenges with infant and young child feeding (IYCF) on social media, this descriptive research aimed to gather empirical evidence of challenges that parents in Ireland experienced with IYCF during the COVID-19 pandemic.

## Methods

### Study design and data collection

A cross-sectional survey was conducted using a self-administered online questionnaire; thus, data were collected at a single point in time. Online surveys have both pros and cons^(25)^; arguably, the main disadvantage is the potential for bias, particularly selection bias as your sample is strongly influenced by where the survey link is shared and who then shares it with others. The sample is also limited to those with access to the internet and sufficient literacy to complete the questionnaire. Despite these known disadvantages, we chose to conduct an online survey as it was our only available option.

The first restrictions, as a result of the first confirmed case of COVID-19 in the Republic of Ireland, were announced on 12th March 2020, with the closure of schools and childcare facilities, followed closely by the closure of almost all businesses, venues and amenities on 24th March^(26)^. On 27th March, the first stay-at-home order was issued that banned all non-essential travel (outside a 2 km radius from home) and contact with other people^(26)^. The first nationwide lockdown (period of severely restricted movement) remained in place until June 2020, when restrictions were gradually lifted in a phase approached until 10th of August^(26)^. Throughout the article, this period from March to June is simply referred to as ‘lockdown’. It was during this period that data collection occurred.

The questionnaire was open online from 11th May 2020 until 12th June 2020 to collect data during the lockdown period. The questionnaire was available for 1 month only as we wanted to collect data before lockdown restrictions completely eased. The time available for data collection determined our sample size; we had anticipated *a priori* that we would receive 500–1000 responses based on previous survey research we had conducted.

### Eligibility criteria

Eligible participants were over the age of 18, lived in Ireland and were the primary caregiver of a child under the age of 2.

### Recruitment of participants

The link to the questionnaire was posted by the study authors on Twitter and Facebook; the admin teams of various parenting support-group social media pages were contacted to advertise the survey, such as platforms for information on infant feeding and breast-feeding support groups. Charities and infant and neonatal health services were contacted to share the questionnaire with their contacts. Thus, a convenience sample was recruited, and it was not possible to calculate a response rate. This recruitment strategy likely influenced the profile of our sample, and we must acknowledge the likelihood of selection bias here.

### Questionnaire development

The questionnaire was developed by the study authors to directly address our research aim, and sections exploring the following concepts were included: demographic characteristics, infant feeding history (using previously published questions that have been assessed for validity and reliability^(27)^), experiences of breast-feeding during lockdown and experiences of formula feeding during lockdown with a focus on challenges experienced by parents. The questionnaire was developed using the SurveyMonkey platform and was repeatedly tested by the authors and other experts in the field to determine face validity and appropriate ordering of questions and functioning of skip patterns (i.e. the technical functionality of the online questionnaire). Two Irish volunteer infant-feeding support/advocacy groups provided input into the final version of the questionnaire.

Most of the questions were closed-ended, though participants were always allowed to select an option titled ‘Other’ and provide more information if the response options provided by the investigators were not considered appropriate. Some questions included a ‘Not applicable’ response option also. Though the focus of the study was predominately quantitative, a small number of qualitative questions were asked, specifically asking participants ‘what would have made your experience with breast-feeding/formula feeding easier during the Coronavirus crisis?’

### Ethical considerations

The first page of the questionnaire described the study and informed participants that we would not be collecting identifying information, that their participation was voluntary and that there was no incentive for participating. Participants were then asked, ‘Do you consent to proceed with this questionnaire?’ Those who selected ‘Yes’ progressed to the next page. This research received ethical approval from the Technological University Dublin Research Ethics Committee (REC-19-160).

### Data cleaning

There were 771 responses to the questionnaire. One respondent was removed from the sample as they indicated that they no longer had any children under 18 living at home and were, thus, ineligible. Six further respondents were removed from the sample as they indicated that their child was aged over 2 years. Finally, nineteen participants who indicated that their child was in the Neonatal Intensive Care Unit (NICU) during lockdown were removed as the NICU experience is not the focus of this article.

### Data analysis

A descriptive quantitative data analysis was conducted in SPSS, version 26. Continuous values are presented as mean (SD), and categorical values are presented as *n* (%) throughout. Qualitative data were managed using NVivo software, version 12. For questions that had an ‘other’ option along with investigator-initiated responses, participant-initiated responses were read and categorised into groups. Open-ended questions about things that would have made breast-feeding or formula feeding easier during the crisis were analysed using Reflexive Thematic Analysis^(28)^ by EJOS. Phase 1 of the analysis involved repeated reading of the responses on the Excel file on which they were originally downloaded to enable familiarisation. The data were then imported into NVivo and were coded; predominantly, semantic codes were attributed to small segments of the data, while consistently reflecting on the focus of the question, which was challenges experienced with IYCF during the pandemic (Phase 2). Initially, no overt attempt was made at latent analysis, though this occurred naturally through familiarisation with the data and some latent codes were applied at this stage. These codes were then reviewed and revised; codes that were similar were combined and code names modified as necessary. The data were revised and coded again, with a focus on latent codes and making connections between codes. During the analysis process, an analytic memo was composed where ideas regarding codes that were similar, those that may be categorised together and potential themes were noted (Phase 3). Next, themes were developed by reviewing potential themes outlined in the memo and deciding whether the proposed options had enough data to be considered a theme, whether the data were meaningful when combined and whether the themes developed provided what was considered a meaningful description of the participants’ responses. The analysis was discussed with the second author as it progressed, but all qualitative analysis was completed by one researcher: a female nutritionist in her mid-late thirties who has two children who were both breastfed, one during the pandemic in Ireland. The primary analyst’s lived experience of breast-feeding through the pandemic enabled her to analyse the data from the position of an insider.

The results are presented below in the order in which the questions appeared in the survey; the experiences of those who breastfed are presented first, followed by those who formula fed. Throughout, we first discuss the experiences of those whose babies were born during the pandemic, followed by those whose baby had been born before the pandemic began. We split our sample in this way because during our initial exploratory analyses – particularly of the qualitative data – it became apparent that the experiences of those who had their baby during the pandemic were different to those whose babies were born before the pandemic began.

## Results

### Participants

There were 771 responses to the questionnaire and, after removing the twenty-six described above, the final sample consists of 745 respondents: 135 who had their baby during the COVID-19 crisis and 610 of whom had already had their child before the pandemic began. The demographic characteristics of the sample are presented in Table 1; over 99 % of the respondents were mothers and most were married or had a partner. In general, the sample was well-educated with most participants reporting to have third-level education. Approximately 70 % of participants lived in an urban location (inner city, suburban, large regional town, small regional town), with 30 % living in a rural location (country village, countryside). In 2019, 63 % of the Irish population lived in an urban location, and 37 % lived in a rural location^(29)^. A very small proportion of the sample had a medical card (5·6 %; *n* 34); in Ireland, people below a certain income receive a medical card and are eligible to receive certain medical services free of charge. In 2019, 19·3 % of those in the general population aged 25–34 years and 22·1 % of those aged 35–44 years had a medical card^(30)^. In both the quantitative and qualitative analyses, we observed differences in experiences reported by those whose babies were born during the pandemic and those whose babies were born before the pandemic. This was most noticeable among breast-feeding mothers. Of the infants who were born during the pandemic (*n* 135), the mean age was 1·8 months (sd 1·1), while the mean age of the infants born prior to the pandemic (*n* 610) was 11·2 months (sd 6·1); results for these two groups are presented separately below, where appropriate.

**Table 1:** Subject characteristics (*n* 745)

Characteristic	Baby born during lockdown (*n* 135)		Had child prior to lockdown (*n* 610)	
	*n* or mean	% or sd	*n* or mean	% or sd
Respondent’s age (years)*	34·8	3·0	35·2	4·1
Child’s age (months)*	1·8	1·1	11·2	6·1
Respondent’s relationship to child				
Mother	134	99·3	607	99·4
Father	1	0·7	3	0·6
Feeding mode at the time of survey				
Breast milk only	101	74·8	91	14·9
Formula only	16	11·9	17	2·8
Breast milk and formula	18	13·3	19	3·1
Breast milk and solid food			303	49·7
Formula and solid food			76	12·5
Breast milk, formula and solid food			36	5·9
Cows’ milk and solid food			58	9·5
Missing			10	1·6
Geographic location				
Inner city	11	8·1	40	6·6
Suburban	58	43	254	41·6
Large regional town	12	8·9	74	12·1
Small regional town	18	13·3	61	10·0
Country village	15	11·1	63	10·3
Countryside	21	15·6	118	19·3
Education				
Third-level or greater	124	93·2	568	93·6
Secondary/post-leaving cert	9	6·8	39	6·7
Marital status				
Married/partner	135	100	597	97·2
Single/separated/divorced	0		11	1·8
Work status				
Maternity leave	127	94·1	244	40·3
Working from home	3	2·2	161	26·6
Caring for children at home	3	2·2	70	11·6
Not working for money	2	1·5	63	10·4
Working outside the home	0		61	10·1
Full-time student	0		6	1
Lost job due to COVID-19 (yes)	2	1·5	38	6·2
Receiving pandemic unemployment payment (yes)	0		35	5·7
Has medical card (yes)	4	3	34	5·6

*The values provided are the mean and the standard deviation, as opposed to n and %.

### Experiences of those who breastfed during lockdown

Over 95 % of the total sample ever breastfed (*n* 711), and 595 had experience of breast-feeding during the pandemic: 125 who had babies during the pandemic and 470 whose babies were born before the pandemic. The COVID-19 pandemic appears to have induced some mothers to breastfeed for longer than they had originally intended. Among the breast-feeding mothers who had their baby during the pandemic, 23·2 % (*n* 29/125) reported changing their breast-feeding behaviour during lockdown. Most of these (*n* 25/29; 86·2 %) decided to stop breast-feeding later than they had originally planned because of the virus. Similarly, among those breast-feeding mothers who had their child prior to the pandemic, 31 % (*n* 146/470) reported changing their breast-feeding behaviour during lockdown. Again, most reported that they decided to stop breast-feeding later than they had originally planned (*n* 124/146; 85·0 %).

#### Challenges experienced by those who breastfed during lockdown

Compared with those whose babies were born before the pandemic, a greater proportion of those whose babies were born during the pandemic experienced challenges with breast-feeding; over 86 % of those who had their babies during the pandemic reported experiencing at least one challenge with breast-feeding, and only 14 % experienced no challenges (Table 2). The main challenges reported by this group were lack of access to support groups (72·8 %) and lack of access to in-person, one-to-one breast-feeding assistance or support (68·8 %). Similarly, being unable to access breast-feeding support groups and in-person support were the most reported challenges among those whose babies were born prior to the pandemic; however, a larger proportion of this group (48·5 %) experienced no challenges with breast-feeding during the COVID-19 crisis.

**Table 2:** Breast-feeding challenges experienced during the lockdown (*n* 595)

Challenge*	Baby born during lockdown and breastfed during lockdown (*n* 125)		Had child prior to lockdown and breastfed during lockdown (*n* 470)	
	*n*	%	*n*	%
No challenges	17	13·6	228	48·5
Unable to access breast-feeding-support groups	91	72·8	143	30·4
Unable to access in-person, one-to-one breast-feeding assistance or support	86	68·8	68	14·5
Concern about stress decreasing your milk supply	38	30·4	57	12·1
Been encouraged to stop breast-feeding by others	16	12·8	46	9·8
Decrease in supply	16	12·8	20	4·3

* Respondents were able to select more than one response.

Those who selected ‘other’ in response to the question ‘Have you experienced any of the following challenges with breast-feeding during the coronavirus crisis?’ and provided additional information (*n* 44) described similar challenges regardless of when their baby was born. Most reported was fear of being separated from their baby if they got sick or if they had to return to work (*n* 18). Several also described issues related to mastitis (*n* 8), for example, one respondent stated, ‘Getting mastitis because I didn’t go to GP when I first felt unwell as I felt we should not be going to the GP unless we had coronavirus’. Concerns regarding increased frequency of feeding and increased demands on the mother were also reported (*n* 6), for example, one respondent stated, ‘Just challenged by tandem feeding two children all day when working from home and tired’.

#### What would have made breast-feeding easier during the COVID-19 crisis

There were 235 responses to the qualitative question: ‘[d]escribe anything that would have made your experience with breast-feeding easier during the Coronavirus crisis’, with thirty-one indicating some variation of ‘nothing’ or ‘not applicable’. Reflexive thematic analysis of the remaining responses (*n* 129 who had a baby prior to lockdown and *n* 75 whose baby was born during lockdown) resulted in the generation of three themes: (1) there was insufficient access to professional and lay breast-feeding support; (2) more information about breast-feeding during a pandemic was needed and (3) COVID-19 either made breast-feeding/parenting harder or easier, depending on the individual. In agreement with the quantitative findings, those who had their baby during the pandemic expressed greater concern about the lack of breast-feeding-related support than other themes described, and those who already had a baby and who felt that they had breast-feeding established acknowledged that things would have been more difficult for them if their baby had been born during the pandemic.

##### Theme 1: Insufficient access to professional and lay support

Professional support was described as coming from International Board-Certified Lactation Consultants, midwives, public health nurses and GPs, and lay support was described as support from volunteer breast-feeding organisations and family/friends. Many described a strong preference for in-person support, especially as breast-feeding is such a ‘hands-on thing’. However, phone or online support was described as an alternative if in-person support was not possible and was often described as being better than nothing.

‘Access in person to support groups/lactation consultant etc to check latch/positioning and give feedback. Taking videos/video calls are not exactly the same thing!’

‘Access to a support group, even online. There are times when we’ve really struggled and I think the support would have been very beneficial’.

Mothers described needing professional support for clinical issues that they felt couldn't be addressed over the phone. Mothers described concerns related to tongue tie, difficulty latching, their baby’s weight, and mastitis. Some felt that additional support in the hospital may have mitigated the issues created by Public Health Nurse clinics closing: ‘Everything is being cancelled … they should have provided much more support in hospital’. Mothers appeared to feel very let down by this lack of healthcare and some described being anxious and very emotionally distressed, describing feeling like they are ‘failing’ or ‘very alone’.

‘Access to tongue tie release. I have suffered for months because of it. Have been stressing that child has not been getting what he needs because of tongue tie. Access to lactation consultant might have helped more’.

‘No contact with any healthcare professional from day 5 until infant was 9 weeks old hence no weight checks as 2 week and 6 week GP check cancelled due to virus. Weight check would have been reassuring and given more confidence with beeastfeeding (sic)’.

‘…feel can’t make contact with Doctor as didn’t want to go to surgery. Eventually had to do online GP consult as clearly had developed mastitis but couldn’t access my own GP as she doesn’t have video access’

Many mothers whose babies were born before lockdown acknowledged that they had established breast-feeding already and they noted that they would have found breast-feeding a lot harder if they were a new mother:

‘Im an experienced breastfeeder and I really feel for new mams at this time of crisis. Breastmilk is so important for a childs health but more so with a dangerous virus around. Not having support at the early stages could be detrimental to a new mothers bf journey’.

‘My baby was approx 9·5 months old when the crisis started so breastfeeding was well established by then. …. I can only imagine how difficult it must be at the moment to get to grips with breastfeeding without being able to access any supports during the crisis’.

##### Theme 2: More information about breast-feeding during a pandemic was needed

Some respondents noted that information or a ‘good resource’ would be helpful for breast-feeding-related queries, given that breast-feeding clinics were closed. However, others mostly discussed information related to coronavirus. Specifically, respondents wanted information about whether the virus could be passed to their child through breast milk, whether it was safe to breastfeed with coronavirus and, most saliently, assurances that mothers and babies would not be separated if the mother had to be hospitalised with coronavirus:

‘Clear info on if the virus passed into breast milk’

‘Earlier direction on what to do if you got sick and how you should feed your child if that happens’

‘I think the initial fear of catching the virus, and being separated from my baby was the most stressful part.…. I think if there was information given about what would happen if I was in hospital- could my baby stay with me, if not could someone collect my pumped milk. It would have been a relief and caused less worry’.

##### Theme 3: COVID-19 either made breast-feeding/parenting harder or easier, depending on the individual

Many respondents described breast-feeding and parenting during the pandemic as ‘difficult’ or ‘isolating’ and described needing help with childcare for other children so the mother could focus on the baby. Many felt that reducing the demands on a mother’s time would be helpful as they described juggling multiple demands at home such as working, home-schooling and breast-feeding:

‘Juggling working from home and having 3 children to also look after is exhausting and stressful. I think it has impacted my supply’.

‘My son is definitely feeding more during the day as I am home ALL the time!! I hope he will adjust when I do go back to the workplace’

‘I feel sore, tired and alone! Other than my husband there is no one to help with the baby so I can take a break/relax/sleep which might help supply and pain’.

However, there were some who reported that their breast-feeding experience was easier during lockdown as they were not separated from their baby and could breastfeed on demand: ‘corona virus means I can now be at home and still breast feed my baby and also work which would never been approved otherwise’ and ‘Not going out so feeding is easier on demand’. Many who described things being easy during the pandemic had previous breast-feeding experience:

‘This is my second time breastfeeding and I know I would have struggled much more had this crisis occurred when I was feeding my first baby’

I didn’t require support as this is not my first baby and I am a confident and experienced breastfeeder”.

### Experiences of those who formula fed during lockdown

#### Challenges experienced by those who formula fed during lockdown

Contrary to the findings observed among those who breastfed during lockdown, a greater proportion of those who had their baby prior to the pandemic and formula fed experienced challenges with formula feeding when compared with those who had their baby during the pandemic. Approximately 44 % of those who had a baby during the pandemic experienced no challenges with formula feeding during lockdown, compared with just 15·9 % of those who had their baby prior to the pandemic (Table 3). Regardless of when their baby was born, the most frequently reported challenges with formula feeding were fearing that there would be formula shortages, purchasing additional formula in case of future shortages and experiencing difficulties purchasing formula due to shortages (Table 3).

**Table 3:** Formula-feeding challenges experienced during the lockdown (*n* 227)

Challenge*	Baby born during lockdown and formula fed during lockdown (*n* 57)		Had child prior to lockdown and formula fed during lockdown (*n* 170)	
	*n*	%	*n*	%
No challenges	25	43·9	27	15·9
Been afraid that there would be formula shortages	28	49·1	112	64·7
Purchased extra formula at any time in case of future shortages	18	31·6	95	55·9
Been unable to purchase formula due to shortages	12	21	57	33·5
Been unable to purchase your preferred brand due to shortages	3	5·3	28	16·4
Been unable to purchase formula due to lack of money	1	1·8	6	3·5
Introduced solid food earlier than you otherwise would have due to formula shortage or fear of shortage	0		3	1·8
Been unable to make up infant formula due to lack of electricity	0		2	1·2
Used more water and fewer scoops of formula in order to stretch how long formula would last	0		1	0·6
Been unable to access formula from a food bank or charity	0		0	

* Respondents were able to select more than one response.

Of those who selected ‘other’ in response to the question ‘Have you experienced any of the following challenges with formula feeding during the coronavirus crisis?’ and provided additional information (*n* 7), three described buying additional formula for various reasons (e.g. to reduce the number of trips to the grocery store or because smaller volume bottles were not available so larger volume bottles were purchased leading to waste). Three described trying or wanting to limit the amount of formula provided and one stopped feeding formula and switched to cows’ milk.

#### What would have made formula feeding easier during the COVID-19 crisis

There were fifty-six responses to the qualitative question: ‘[d]escribe anything that would have made your experience with formula feeding easier during the Coronavirus crisis’, with eight indicating some variation of ‘nothing’ or ‘not applicable’. Reflexive thematic analysis of the remaining forty-eight responses indicated that respondents’ concerns were similar regardless of whether their baby was already born when the crisis started or was born during the crisis, and two themes were generated from the data: (1) consistent access to formula and assurances that there would be no shortages were needed and (2) infant feeding information and support (including financial) were needed.

##### Theme 1: Consistent access to formula and assurances that there would be no shortages were needed

Most responses to this question focused on logistics around the availability of formula in shops, a need for assurances that there would be no shortages, with some focusing on the need for affordability. Many comments described a need for a consistent and reliable supply of formula:

‘Having enough supply in shops so I didn’t have to travel to the next town to get it’

‘I did the “right” thing and didn’t stockpile formula but then felt punished for this when the supermarkets ran out. It was stressful trying to find it. Managed to get supermarket own brand to tide us over but baby’s tummy wasn’t used to it and stools were loose and more frequent and baby was windier too’

Some mothers described other people ‘bulk buying’ or ‘stocking up’ on infant formula, and a couple noted that seeing evidence of this on social media was stressful. Solutions suggested to this including placing limits on the amount of formula one person could buy in a shop:

‘The supermarkets should have stopped people buying more than 2 boxes of formula per purchase. I witnessed people buying multiple (8+) boxes of different types of formula/ages ranges’.

In general, participants wanted more stock of formula available, and a small number of participants suggested that assurances from public health officials regarding the supply of formula would have been helpful: ‘If public health officials guaranteed supply of formula during crisis’.

Finally, not having to use formula at all was suggested by five participants as something that would have made their experience easier, for example, one stated ‘It would have been easier if I was still breastfeeding’.

##### Theme 2: Infant feeding information and support (including financial) were needed

Some people described simply needing any support at all but also needing ‘unbiased’ ‘guidance’ around quantities of infant formula to use, weaning from the breast to infant formula or their baby’s weight. This was considered particularly important during the pandemic:

‘Contact from the public health nurse as she has been a no show since I had the baby really.’

‘More information needed on formula feeding in general, safety and preparation. Especially needed in times of extreme stress and anxiety’.

A few participants commented on the affordability of formula with one stating that ‘universal pricing’ would mean not having to travel further to cheaper shops. One participant described the cost of formula as a ‘burden’ on her family.

## Discussion

In this survey of caregivers of infants and young children, we explored the experiences of parents – predominantly mothers – in Ireland with breast-feeding and formula feeding during a national emergency. Our findings have highlighted that parents experienced multiple challenges with infant feeding during the COVID-19 crisis, which caused additional stress and anxiety for them. Many parents, regardless of whether they were breast-feeding or formula feeding, reported challenges. Although infancy can be a challenging time for caregivers in general, and issues related to infant feeding often cause anxiety, we feel that the issues highlighted by parents in this study were either directly related to COVID-19 or considerably exacerbated by societal changes that were introduced to curb the spread of the virus. Breast-feeding mothers were stressed and concerned about a lack of support and assistance with breast-feeding and parents who were using formula reported anxiety about a consistent and affordable supply of formula being available. Whether or not there was a true issue with supply of formula, the actions of those who feared a shortage – namely stocking up on formula – may have created a shortage for others. Arguably, the most affected in this study were those who gave birth during the lockdown period and wished to breastfeed. It was generally acknowledged by respondents to this survey that getting breast-feeding established initially is difficult, and this would be most difficult to do with limited support and added stress. On top of challenges with infant feeding, many participants in this survey reported experiencing increased demands on their time, as they had to homeschool older children and/or work from home while also feeding and caring for a younger child. The increased demands on women and mothers reported in this survey have also been described by other researchers^(31–33)^.

Our findings are in agreement with those of investigators who have conducted similar survey-based research in Belgium^(34)^, Australia^(5)^, the UK^(35)^ and Canada^(36)^. In Belgium, a large proportion of women felt they breastfed longer because staying at home facilitated breast-feeding and they wanted to minimise risk of coronavirus infection, but their access to breast-feeding support was severely limited^(34)^. Similarly, Australian mothers were concerned about the inability to access face-to-face services (because groups were cancelled or they did not want to attend healthcare facilities for fear of contracting COVID-19), and several reported stress and anxiety associated with isolation^(5)^. Mothers in the UK also reported a lack of breast-feeding support during lockdown, particularly in-person support, and also a lack of social support due to the absence of mother-and-baby groups^(35)^. Inadequate breast-feeding support is concerning; lack of professional breast-feeding support was the most common reason given by women in one UK study for stopping breast-feeding^(37)^. In Canada, over a quarter of the breast-feeding parents surveyed reported challenges with breast-feeding during their State of Emergency; two-thirds stated that the challenges were specifically related to the State of Emergency and the inability to access lactation consultants or public health nurses^(36)^. Fry and colleagues also reported that several participants experienced stress related to issues with formula access, either there were shortages of formula – especially specialised formulae – or formula in general was not affordable^(36)^. Concerns regarding shortages of formula were also reported by a sample of mothers who participated in qualitative interviews in the USA^(38)^, which encouraged some to continue breast-feeding. Similarly, Hull and colleagues reported women wanting to continue breast-feeding during the pandemic as they were afraid they might not be able to access infant formula^(5)^. It is worth noting that, in our sample, a larger proportion of parents whose babies were born prior to the pandemic reported concerns about formula shortage. This may be because, having older babies than those whose babies were born during the pandemic, they spent more time in supermarkets and were observing the consequences of shoppers purchasing foods and other items in bulk. This may have made them more concerned about the supply of formula.

Other qualitative research conducted during the pandemic largely echoes our findings and those of the researchers described above. Among women who participated in a recurrent cross-sectional qualitative study in the UK, limited access to both professional and lay support was reported^(39)^. In particular, lack of face-to-face support was problematic and there were concerns around attending healthcare facilities due to risk of contracting COVID-19^(39)^. In Spain, reduced access to professional support was reported as a challenge by women who participated in qualitative interviews, but in general social restriction measures were viewed positively as they allowed mothers more time with their babies and limited visitors, which facilitated breast-feeding^(40)^. Brown and Shenker also reported that nearly 42 % of their sample reported that lockdown had an overall positive impact on their breast-feeding experience. Participants in their study reported having nowhere to go, having more privacy, fewer visitors and being able to feed on demand as positive impacts of lockdown^(37)^, which was also noted in the USA^(38)^. Cohen and Botz described the isolation as ‘a blessing or a curse depending on the situation’^(33)^. Some of their participants reported that a limited number of visitors allowed them to breastfeed whenever they want without feeling the need to shield themselves, while others felt isolated and missed the support they would otherwise have had from family members, friends or a ‘village of mamas’^(33)^. The differing impacts – positive and negative – on women’s breast-feeding experiences during lockdowns have also been described in a narrative review of twelve studies conducted internationally during the COVID-19 pandemic^(41)^. Positive aspects centred on women having more time to be at home with their babies and being able to breastfeed more, whereas, in line with the present findings, the negative aspects mainly related to restrictions on professional support and social support^(41)^.

The nutritional vulnerability of infants and young children, and the potential for malnutrition associated with interrupting breast-feeding or limited access to safe alternatives to breast-feeding, formed the rationale for the development of an IYCF-E guidance document for emergency relief staff and programme managers, first published in 2001 and most recently updated in 2017^(17)^. While this document provides invaluable information, the IFE Core Group also recommend the development of a context-specific preparedness plan for IYCF-E. During emergency situations, it becomes increasingly likely that recommended practices for IYCF will not be followed, due to disrupted access to healthcare, food, water and other resources. Considering this, at the 71st World Health Assembly in 2018, the WHO urged all member states to ‘take all necessary measures to ensure evidence-based and appropriate infant and young child feeding during emergencies, including through preparedness plans…’^(16)^. Unfortunately, Ireland has no such plan in place. It is clear from the experiences of those in our study that there was no coordinated effort to protect infant feeding during lockdown, and infant feeding was not prioritised by the Government of Ireland at the height of this emergency. Having an IYCF-E plan would provide considerable protection against food insecurity and stress for women and families with young children.

In many countries, ‘emergencies’ are natural or climatic emergencies, such as floods, fires, hurricanes, heatwaves and volcano eruptions, or man-made disasters – in particular, wars. However, the COVID-19 pandemic has resulted in widespread emergency situations in countries like Ireland that rarely experience major climatic emergencies. Disaster and emergency situations can and do occur in Ireland; climate-related severe weather events mean things like flooding and snowstorms are becoming more common, leaving families without access to electricity, clean water or local shops from which to purchase formula. Ireland also experiences contamination of local water supplies with some regularity^(42)^, which severely impacts the ability to safely reconstitute formula. During the COVID-19 pandemic, particularly at the beginning, public health messaging from Governmental departments around the importance of breast-feeding – with concomitant resources devoted to protecting and supporting breast-feeding – and the security of supply chains for infant formula may have made infant feeding considerably easier and less stressful for many parents. As such, it is imperative that a comprehensive Irish IYCF-E Plan is developed.

### Strengths and limitations

Given the public health restrictions in Ireland during the data collection period, the use of an online survey was a strength of this study. It helped overcome some of the challenges of contacting caregivers, allowing a large sample to be surveyed. However, caution is needed in terms of generalisation to the Irish population. Like many online surveys, the respondents were older mothers with a high level of education, who had access to the internet. There was also a small proportion of the sample who had a medical card, suggesting that this sample was of higher socio-economic status than the general population. This means that the experiences of those of lower socio-economic status, who may have had different and/or worse challenges, may not be reflected in our data. It is also possible that those caregivers with the most extreme (negative and positive) experiences may have been more likely to participate. A UK study reported that mothers with a lower education, from a minority ethnic background or those with more challenging living circumstances found the impact of lockdown on breast-feeding more challenging and stopped breast-feeding sooner^(37)^. Other studies have described potential ethnic differences in experiences^(39)^. Our sample was highly educated, and we did not collect data on ethnicity. As such, the experiences of some subgroups may not be reflected in our data.

### Conclusion and recommendations

The COVID-19 pandemic was an infectious disease emergency that made IYCF both challenging and stressful for mothers and other caregivers across Ireland, regardless of how they chose to feed their child. This research highlights the need for prioritising breast-feeding support services during emergencies as well as the need for assurances and possibly planning around the supply infant formula nationally. Most importantly, the research highlights the urgent need for an Irish IYCF-E preparedness plan, which would protect the nutritional needs of the most vulnerable members of our population. We recommend that such a plan is developed based on WHO/international recommendations, integrated into existing national emergency-preparedness plans and communicated to all relevant agencies.

## References

[1] Victora CG , Bahl R , Barros AJ et al. (2016) Breastfeeding in the 21st century: epidemiology, mechanisms, and lifelong effect. Lancet 387, 475–490. 26869575 10.1016/S0140-6736(15)01024-7

[2] Creek TL , Kim A , Lu L et al. (2010) Hospitalization and mortality among primarily nonbreastfed children during a large outbreak of diarrhea and malnutrition in Botswana, 2006. J Acquir Immune Defic Syndr 53, 14–19. 19801943 10.1097/QAI.0b013e3181bdf676

[3] Hipgrave DB , Assefa F , Winoto A et al. (2012) Donated breast milk substitutes and incidence of diarrhoea among infants and young children after the May 2006 earthquake in Yogyakarta and Central Java. Public Health Nutr 15, 307–315. 21426621 10.1017/S1368980010003423

[4] Khajehaminian MR , Hosseini Boroujeni SM , Ghanbari V et al. (2020) Breastfeeding in disasters: a reminder for policymakers. J Disaster Emerg Res 2, 110–114.

[5] Hull N , Kam RL & Gribble KD (2020) Providing breastfeeding support during the COVID-19 pandemic: concerns of mothers who contacted the Australian breastfeeding association. Breastfeed Rev 28, 25–35.

[6] Hoang DV , Cashin J , Gribble K et al. (2020) Misalignment of global COVID-19 breastfeeding and newborn care guidelines with World Health Organization recommendations. BMJ Nutr Prev Health 3, 339. 10.1136/bmjnph-2020-000184 PMC7759756 33521544

[7] Stuebe A (2020) Should infants be separated from mothers with COVID-19? First, do no harm. Breastfeed Med 15, 351–352. 32271625 10.1089/bfm.2020.29153.ams PMC7236243

[8] World Health Organization (2020) Breastfeeding and COVID-19: Scientific Brief, 23 June 2020. Geneva: World Health Organization.

[9] Lubbe W , Botha E , Niela-Vilen H et al. (2020) Breastfeeding during the COVID-19 pandemic–a literature review for clinical practice. Int Breastfeed J 15, 1–9. 32928250 10.1186/s13006-020-00319-3 PMC7487446

[10] Liu X , Chen H , An M et al. (2022) Recommendations for breastfeeding during Coronavirus Disease 2019 (COVID-19) pandemic. Int Breastfeed J 17, 28. 35410357 10.1186/s13006-022-00465-w PMC8995694

[11] Salmon L (2015) Food security for infants and young children: an opportunity for breastfeeding policy? Int Breastfeed J 10, 7. 25750657 10.1186/s13006-015-0029-6 PMC4352266

[12] Scherbaum V & Srour ML (2016) The role of breastfeeding in the prevention of childhood malnutrition. World Rev Nutr Diet 115, 82–97. 27198529 10.1159/000442075

[13] Ntambara J & Chu M (2021) The risk to child nutrition during and after COVID-19 pandemic: what to expect and how to respond. Public Health Nutr 24, 3530–3536. 33845938 10.1017/S1368980021001610 PMC8144817

[14] O’Halloran M & Pope C (2020) Panic Buying Will Cause Problems That Do Not Currently Exist, Says Humphreys. In The Irish Times. https://www.irishtimes.com/news/ireland/irish-news/panic-buying-will-cause-problems-that-do-not-currently-exist-says-humphreys-1.4201177 (accessed May 2022).

[15] Guynn J (2020) Baby Formula Shortages Easing After Coronavirus Panic Buying, But Don’t Expect Fully Stocked Shelves for Months. In USA Today. https://eu.usatoday.com/story/money/2020/04/17/coronavirus-shopping-baby-formula-infant-formula-shortage-covid-19/5139317002/ (accessed May 2022).

[16] World Health Assembly (2018) Seventy-first World Health Assembly: Infant and Young Child Feeding. https://apps.who.int/iris/bitstream/handle/10665/279517/A71_R9-en.pdf (accessed May 2022).

[17] IFE Core Group (2017) Infant and Young Child Feeding in Emergencies: Operational Guidance for Emergency Relief Staff and Programme Managers: Emergency Nutrition Network. https://www.ennonline.net/operationalguidance-v3-2017 (accessed August 2022).

[18] Gribble K , Peterson M & Brown D (2019) Emergency preparedness for infant and young child feeding in emergencies (IYCF-E): an Australian audit of emergency plans and guidance. BMC Public Health 19, 1278. 31610779 10.1186/s12889-019-7528-0 PMC6792236

[19] WBTi (2016) World Breastfeeding Trends Initiative UK Report. https://ukbreastfeeding.org/wbtiuk2016 (accessed May 2022).

[20] Zakarija-Grković I , Cattaneo A , Bettinelli ME et al. (2020) Are our babies off to a healthy start? The state of implementation of the Global strategy for infant and young child feeding in Europe. Int Breastfeed J 15, 51. 32493416 10.1186/s13006-020-00282-z PMC7271477

[21] Department of Defence & Office of Emergency Planning (2020) Strategic Emergency Management: National Structures and Framework. https://assets.gov.ie/90681/71eaf4b4-3c20-488d-b443-620e57a51c2b.pdf (accessed May 2022).

[22] Department of Housing, Local Government and Heritage (2005) A Framework for Major Emergency Management. https://assets.gov.ie/180183/5dca2e44-350b-492a-bdc3-e5b1135a918d.pdf (accessed May 2022).

[23] Panda S , O’Malley D , Barry P et al. (2021) Women’s views and experiences of maternity care during COVID-19 in Ireland: a qualitative descriptive study. Midwifery 103, 103092. 34325384 10.1016/j.midw.2021.103092 PMC8582075

[24] Loughlin E & Bowers S (2021) Half of Babies Missing Health Checks due to Covid-19. In Irish Examiner. https://www.irishexaminer.com/news/arid-40261736.html (accessed May 2022).

[25] Ball HL (2019) Conducting online surveys. J Hum Lact 35, 413–417. 31084575 10.1177/0890334419848734

[26] Kennelly B , O’Callaghan M , Coughlan D et al. (2020) The COVID-19 pandemic in Ireland: an overview of the health service and economic policy response. Health Policy Technol 9, 419–429. 32923355 10.1016/j.hlpt.2020.08.021 PMC7480279

[27] O’Sullivan EJ & Rasmussen KM (2017) Development, construct validity, and reliability of the questionnaire on infant feeding: a tool for measuring contemporary infant-feeding behaviors. J Acad Nutr Diet 117, 1983–1990. 28676229 10.1016/j.jand.2017.05.006

[28] Braun V & Clarke V (2022) Thematic Analysis: A Practical Guide: London: Sage.

[29] Central Statistics Office (2019) Urban and Rural Life in Ireland, 2019. https://www.cso.ie/en/releasesandpublications/ep/p-urli/urbanandrurallifeinireland2019/agesexandgeographicaldistribution/ (accessed August 2023).

[30] Central Statistics Office (2020) Ireland’s UN SDGs 2019 – Report on Indicators for Goal 3 Good Health and Well-Being. https://www.cso.ie/en/releasesandpublications/ep/p-sdg3/irelandsunsdgs2019-reportonindicatorsforgoal3goodhealthandwell-being/healthcare/ (accessed August 2022).

[31] Fisher AN & Ryan MK (2021) Gender inequalities during COVID-19. Group Process Intergr Relat 24, 237–245.

[32] Power K (2020) The COVID-19 pandemic has increased the care burden of women and families. Sustain Sci Pract Policy 16, 67–73.

[33] Cohen M & Botz C (2022) Lactation in quarantine: the (in) visibility of human milk feeding during the COVID-19 pandemic in the United States. Int Breastfeed J 17, 1–22. 35313894 10.1186/s13006-022-00451-2 PMC8935117

[34] Ceulemans M , Verbakel JY , Van Calsteren K et al. (2020) SARS-CoV-2 infections and impact of the COVID-19 pandemic in pregnancy and breastfeeding: results from an observational study in primary care in Belgium. Int J Environ Res Public Health 17, 6766. 32957434 10.3390/ijerph17186766 PMC7559009

[35] Vazquez-Vazquez A , Dib S , Rougeaux E et al. (2021) The impact of the Covid-19 lockdown on the experiences and feeding practices of new mothers in the UK: preliminary data from the COVID-19 New Mum Study. Appetite 156, 104985. 33038477 10.1016/j.appet.2020.104985 PMC7538871

[36] Fry HL , Levin O , Kholina K et al. (2021) Infant feeding experiences and concerns among caregivers early in the COVID-19 state of emergency in Nova Scotia, Canada. Matern Child Nutr 17, e13154. 33619906 10.1111/mcn.13154 PMC7995067

[37] Brown A & Shenker N (2021) Experiences of breastfeeding during COVID-19: lessons for future practical and emotional support. Matern Child Nutr 17, e13088. 32969184 10.1111/mcn.13088 PMC7537017

[38] Snyder K & Worlton G (2021) Social support during COVID-19: perspectives of breastfeeding mothers. Breastfeed Med 16, 39–45. 33372829 10.1089/bfm.2020.0200

[39] Jackson L , De Pascalis L , Harrold JA et al. (2021) Postpartum women’s experiences of social and healthcare professional support during the COVID-19 pandemic: a recurrent cross-sectional thematic analysis. Women Birth 5, 511–520. 10.1016/j.wombi.2021.10.002 PMC8553649 34756734

[40] Rodríguez-Gallego I , Strivens-Vilchez H , Agea-Cano I et al. (2022) Breastfeeding experiences during the COVID-19 pandemic in Spain: a qualitative study. Int Breastfeed J 17, 11. 35193625 10.1186/s13006-022-00453-0 PMC8861604

[41] Pacheco F , Sobral M , Guiomar R et al. (2021) Breastfeeding during COVID-19: a narrative review of the psychological impact on mothers. Behav Sci 11, 34. 33799384 10.3390/bs11030034 PMC7999784

[42] Environmental Protection Agency (2022) Drinking Water Quality in Private Group Schemes and Small Private Supplies 2020. https://www.epa.ie/publications/compliance--enforcement/drinking-water/annual-drinking-water-reports/DWQinPrivateGroupWaterSupplies-2022-02-21.pdf (accessed August 2022).

